# LncRNA WT1-AS over-expression inhibits non-small cell lung cancer cell stemness by down-regulating TGF-β1

**DOI:** 10.1186/s12890-020-1146-6

**Published:** 2020-04-29

**Authors:** Xueqin Jiang, Jiong Wang, Lei Fang

**Affiliations:** Department of Geriatric Respiratory and Critical Care, the First Affiliated Hospital of Anhui Medical University, No. 218 Jixi Road, Hefei City, Anhui Province 230022 People’s Republic of China

**Keywords:** Non-small cell lung cancer, lncRNA WT1-AS, TGF-β1, Stemness

## Abstract

**Background:**

LncRNA WT1-AS is a recently identified potential tumor suppressor in gastric cancer. This study mainly explored the role of WT1-AS in non-small cell lung cancer (NSCLC).

**Methods:**

WT1-AS and TGF-β1 mRNA in two types of tissues of 74 NSCLC patients were detected by performing RT-qPCR experiments. WT1-AS and TGF-β1 expression vectors were established using the pcDNA3.1 vector. Protein concentration was measured by BCA assay. Mean values in this study were calculated using the data of three biological replicates of each experiment.

**Results:**

We found that WT1-AS was down-regulated, while TGF-β1 was upregulated in NSCLC tissues. Survival analysis showed that low levels of WT1-AS expression predicted poor survival of NSCLC patients. WT1-AS and TGF-β1 were inversely correlated in NSCLC tissues. Over-expression experiments revealed down-regulated TGF-β1 after WT1-AS over-expression, while TGF-β1 over-expression failed to affect WT1-AS. WT1-AS over-expression resulted in inhibited cancer cell stemness. TGF-β1 over-expression played an opposite role and attenuated the effects of TGF-β1 over-expression.

**Conclusion:**

Therefore, WT1-AS over-expression may inhibit non-small cell lung cancer cell stemness by down-regulating TGF-β1.

**Trial registration:**

The First Affiliated Hospital of Anhui Medical University Ethics committee approved this study (AHMU20101009).

## Background

During the past several decades, the incidence of lung cancer continually increased and this disease gradually becomes a leading cause of cancer-related mortalities in many countries of the world, such China and the United States [1, 2]. In the United States, lung cancer affects more than 1.7 million new cases and causes 0.6 million deaths every year [2]. It is generally believed that more than 90% of deaths in cancer patients are caused by cancer metastasis [3], which is common in lung cancer patients by the time of initial diagnosis [4]. Early diagnosis of lung cancer is challengeable due to the lack of sensitive markers [5, 6]. Therefore, the accurate prognosis may be another option to improve the survival of lung cancer patients.

Non-small-cell lung cancer (NSCLC) is the primary subtype of lung cancer and account for more than 85% of all lung cancer cases. Altered expression of genetic factors, such as tumor suppressors and oncogenes plays pivotal roles in NSCLC [7, 8]. Besides that, non-coding RNAs (ncRNAs), such as its subgroup long ncRNAs (> 200 nt, lncRNAs) also participate in human cancer, including NSCLC by regulating the expression of the protein-coding gene [9]. A particular lncRNA, which is named WT1-AS, attracted our attention. This lncRNA plays tumor-suppressive roles in many types of cancer, such as gastric cancer [10], cervical cancer [11] and hepatocellular carcinoma [12], while its role in NSCLC is unknown. It is known that lncRNAs may have similar functions in different types of cancer. Therefore, WT1-AS may also have tumor-suppressive roles in NSCLC. The present study was carried out to test this hypothesis.

## Methods

### Patients and follow-up

The research subjects of the present study were 42 male and 32 female NSCLC patients (27 to 69 years, 45.3 ± 6.4 years). All the patients were selected in the First Affiliated Hospital of Anhui Medical University between January 2011 and April 2013. Inclusion criteria: 1) newly diagnosed NSCLC patients by histopathological examinations; 2) patients’ therapies were not initiated before admission; 3) patients willing to participate in a 5-year follow-up. Exclusion criteria: 1) recurrent NSCLC (recurrence after treatment); 2) other clinical disorders were observed; 3) any therapies for any diseases were performed within 3 months before admission; 4) who were lost or died of other causes during follow-up. Seventy-four patients included 30 cases of SCC and 44 cases of adenocarcinoma. There were 64 smokers (current or previous) and ten never-smokers. Based on clinical diagnosis data; the 74 patients were grouped into AJCC stage I (*n* = 12), II (*n* = 24), III (*n* = 20) and IV (*n* = 18). The First Affiliated Hospital of Anhui Medical University Ethics committee approved this study. All the 74 NSCLC patients signed informed consent.

### Follow-up

Patients were followed up for 5 years after admission. Follow-up was performed in a monthly manner through telephone and/or outpatient visit. The survival conditions of each patient were recorded.

### Tissues and cells

Before the initiation of therapies, all patients were subjected to diagnosis using techniques like EBUS-TBNA or endoluminal ultrasound-guided FNA. During diagnosis, both cancer (NSCLC) and non-cancer (within about 2 cm around tumors) samples were collected from each patient (about 0.1 g per sample). At least 3 experienced pathologists confirmed all tissues.

NCI-H522 and NCI-H23 human NSCLC cell lines were used in this study. Cells of both cell lines were from ATCC (USA). Cell culture medium was RPMI-1640 medium (10% FBS). Cell culture conditions were 37 °C and 5% CO_2_.

### RT-qPCR

Tissues were ground in liquid nitrogen. Ground tissues, as well as NCI-H522 and NCI-H23 cells, were mixed with Trizol reagent (Invitrogen, USA) to extract total RNAs. Total RNAs were washed with 70% ethonal, followed by DNase I digestion. The digested RNA samples were used as a template to synthesize cDNA using AMV Reverse Transcriptase (Canvax Biotech, USA). KOD SYBR® qPCR Mix (TOYOBO, Japan) was used to prepare all PCR reaction mixtures with 18S rRNA or GAPDH as an endogenous control to analyze the expression of WT1-AS and TGF-β1. ABI PRISM 7500 qRT-PCR machine (Applied Biosystems, USA) was used to perform all PCR reactions. All qPCR reactions were performed three times. Data were normalized using the 2^-ΔΔCT^ method.

### Transient transfection

WT1-AS and TGF-β1 expression vectors were established using the pcDNA3.1 vector, which was provided by Sangon (Shanghai, China). Vectors were subjected to Sanger sequencing to make sure correct gene sequences were obtained. All cell transfections were performed using Lipofectamine 3000 (Clontech, USA). Vectors (10 nM) were transfected into 10^6^ cells. Following studies were performed using cells collected at 24 h after transfections. Control (C, non-transfection) and Negative control (NC, empty vector transfection) were included.

### Flow cytometry

NCI-H522 and NCI-H23 cells were harvested at 24 h after transfections. Cells were subjected to trypsinization. Processed cells were subjected to incubation for 20 min with CD133-PE or IgG1-PE (Control) antibody (Meltenyi Biotec, Germany) at 4 °C. After that, cells were mixed with PBS. FACS Aria system (BD Immunocytometry Systems, USA) was used to capture signals and signals were analyzed using Cell Quest software (Becton Dickinson Ltd). The percentage of CD133+ cells to all cells was calculated and presented.

### Western-blot

NCI-H522 and NCI-H23 cells were mixed with RIPA solution (Thermo Fisher Scientific) to extract total proteins. Protein concentration was measured by BCA assay (Protein concentration was measured by BCA assay. Total proteins were denatured for 10 min at 95 °C and were subjected to electrophoresis using 10% SDS-PAGE gel with 50 μg per lane. After electrophoresis, proteins were transferred to PVDF membranes using a semi dry method. Membranes were blocked for 2 h in 5% non-fat milk at 22 °C, followed by incubation with GAPDH (rabbit polyclonal, ab9485, 1:1400, Abcam) and TGF-β1 (rabbit polyclonal, ab9758, 1:1200; Abcam) primary antibodies for 14 h or overnight at 4 °C. After that, membranes were further incubated with IgG-HRP secondary antibody (goat anti-human, 1:1000, MBS435036, MyBioSource) for 2 h at 22 °C. Signals were developed using ECL (Sigma-Aldrich, USA), and signals were processed using Image J v1.46 software.

### Statistical analysis

Mean values in this study were calculated using the data of 3 biological replicates of each experiment. Differences in expression data between NSCLC and non-cancer tissues were analyzed by performing paired t-test. One-way ANOVA and Tukey test analyzed differences in cell stemness data and gene expression data among different cell transfection groups. Correlations between WT1-AS and TGF-β1 were analyzed by performing linear regression. Based on expression data of WT1-AS in NSCLC tissues, the 74 patients were grouped into high (*n* = 36) and low (*n* = 38) WT1-AS level groups (Youden’s index, Graphpad prism six software). K-M method and log-rank test were used to plot and compare survival curves. Using the same grouping method, the Chi-squared test was to analyze the correlations between patients’ clinical data and WT1-AS and TGF-β1 mRNA expression level. Statistically, the significant level was set to *p* < 0.05.

## Results

### WT1-AS and TGF-β1 mRNA showed opposite expression pattern in NSCLC

**Fig. 1:**
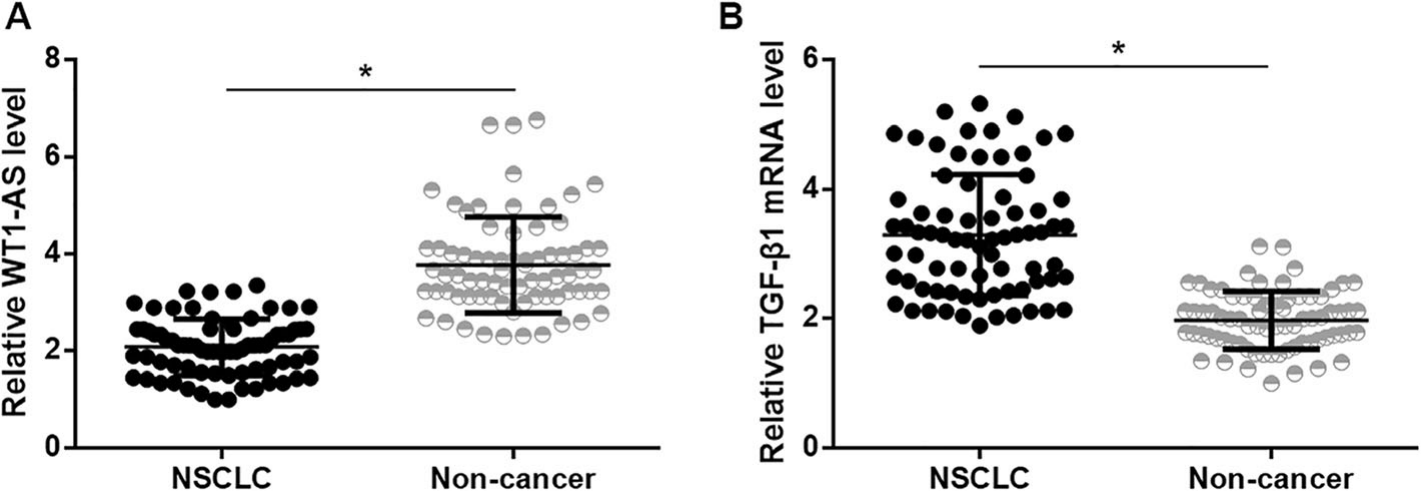
WT1-AS and TGF-β1 mRNA showed opposite expression pattern in NSCLC. Expression data of WT1-AS and TGF-β1 were compared between NSCLC and non-cancer tissues by performing paired t test. It was found that WT1-AS was down-regulated (**a**), while TGF-β1 was upregulated (**b**) in NSCLC tissues than in non-cancer tissues

WT1-AS and TGF-β1 mRNA in two types of tissues (NSCLC and non-cancer tissues) of 74 NSCLC patients were detected by performing RT-qPCR experiments. WT1-AS and TGF-β1 mRNA expression levels were compared between NSCLC and non-cancer tissues by performing a paired t-test. It was found that the expression levels of WT1-AS were significantly lower (Fig. 1a, *p* < 0.05), while expression levels of TGF-β1 were substantially higher (Fig. 1b, p < 0.05) in NSCLC tissues compared to non-cancer tissues. The chi-squared test showed that expression levels of WT1-AS and TGF-β1 mRNA were significantly correlated with the clinical stage (*p* < 0.05), but not subtypes of NSCLC (*p* > 0.05) and smoking history (*p* > 0.05).

### Low levels of WT1-AS in NSCLC tissues predicted poor survival

**Fig. 2:**
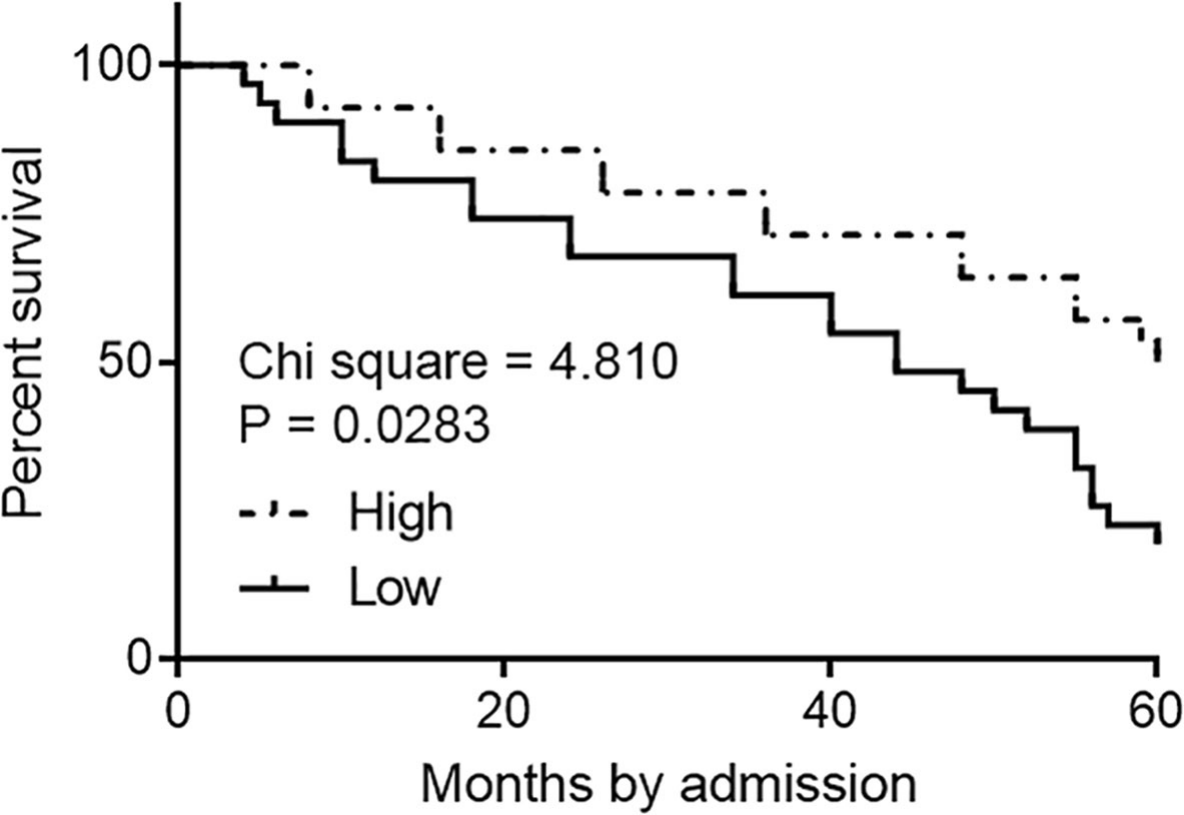
Low levels of WT1-AS in NSCLC tissues predicted poor survival. Survival analysis was performed to analyze the correlation between expression levels of WT1-AS and survival of NSCLC patients. It was observed that low levels of WT1-AS in NSCLC tissues were accompanied by poor survival

The Patients were grouped, followed the method above. No significant differences in treatment strategies were found between the high and low-level groups. Survival curves were plotted and compared using the aforementioned methods. It was observed that the overall survival rate was significantly lower in low WT1-AS level group than in the high WT1-AS level group (Fig. 2).

### WT1-AS down-regulated TGF-β1 in NSCLC cell at both mRNA and protein levels

**Fig. 3:**
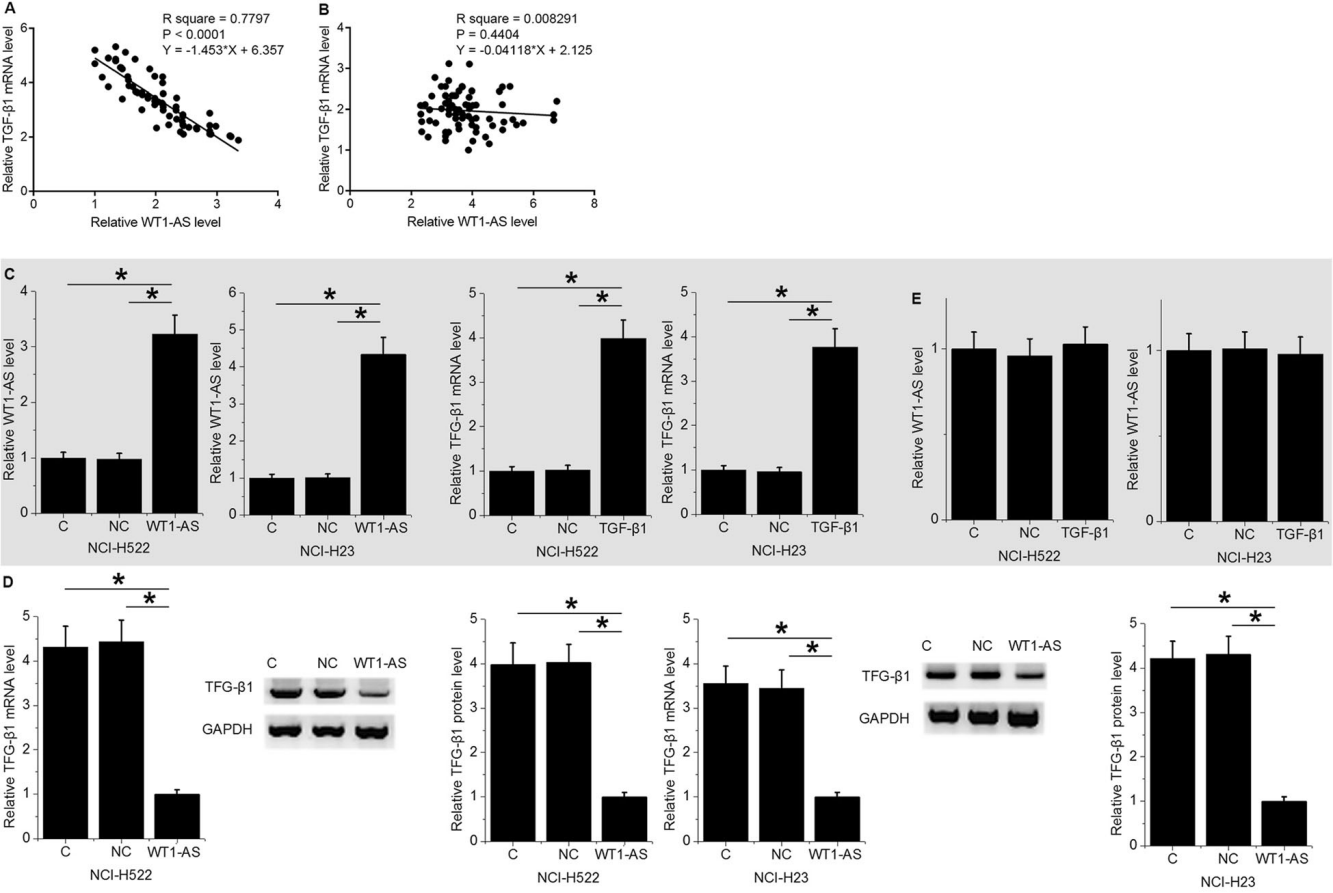
WT1-AS down-regulated TGF-β1 in NSCLC cell at both mRNA and protein levels. Linear regression showed that WT1-AS and TGF-β1 were inversely and significantly correlated in NSCLC tissues (**a**), but not in non-cancer tissues (**b**). WT1-AS and TGF-β1 expression levels were significantly increased at 24 h after transfections comparing to C and NC groups (**c**). Over-expression experiments revealed down-regulated TGF-β1 after WT1-AS over-expression (**d**), while TGF-β1 over-expression failed to affect WT1-AS (**e**), (*, *p* < 0.05)

Results of linear regression showed that, in NSCLC tissues, WT1-AS and TGF-β1 expression levels were inversely and significantly correlated (Fig. 3a). In contrast, no promising correlation between the expression levels of WT1-AS and TGF-β1 in non-cancer tissues was found (Fig. 3b). To further analyze the relationship between WT1-AS and TGF-β1, NCI-H522 and NCI-H23 cells were transfected with WT1-AS and TGF-β1 expression vectors. WT1-AS and TGF-β1 expression levels were significantly increased at 24 h after transfections comparing to C and NC groups (Fig. 3c, *p* < 0.05). In addition, NCI-H522 and NCI-H23 cells with WT1-AS over-expression showed significantly down-regulated TGF-β1 at both protein and mRNA levels comparing to two controls (Fig. 3d, *p* < 0.05). Moreover, NCI-H522 and NCI-H23 cells with TGF-β1 over-expression showed no significantly changed expression levels of WT1-AS (Fig. 3e).

### WT1-AS regulated NSCLC cell stemness through TGF-β1

**Fig. 4:**
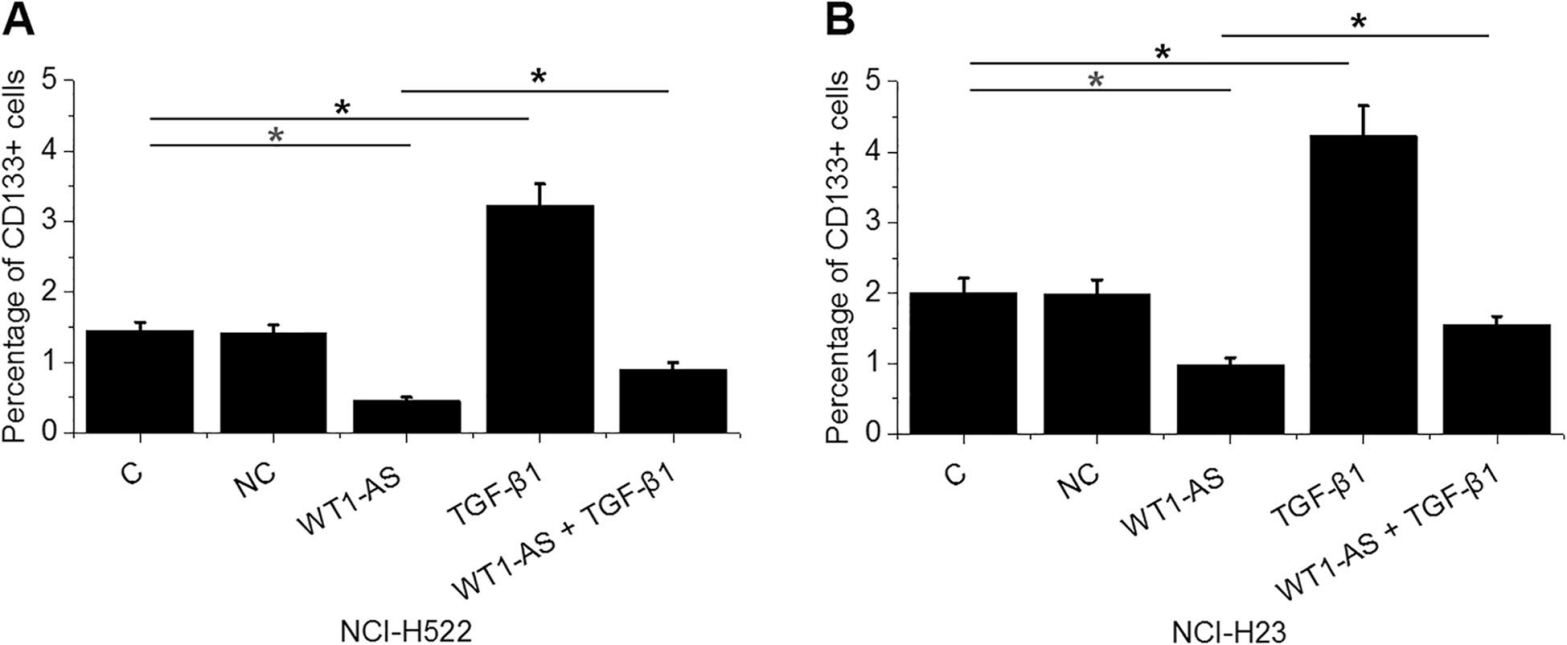
WT1-AS regulated NSCLC cell stemness through TGF-β1. WT1-AS over-expression resulted in inhibited cancer cell stemness (reflected by percentage of CD133+ cells) (**a**). TGF-β1 over-expression played an opposite role and attenuated the effects of TGF-β1 over-expression (*, *p* < 0.05) (**b**)

Comparing to the C and NC groups, WT1-AS over-expression resulted in reduced percentage of CD133+ cells. TGF-β1 over-expression resulted in an increased percentage of CD133+ cells. Compared with cells with WT1-AS over-expression alone, cells with both WT1-AS and TGF-β1 over-expression showed a significantly increased percentage of CD133+ cells (Fig. 4, *p* < 0.05).

## Discussion

We mainly investigated the roles of WT1-AS in NSCLC. We found that WT1-AS was down-regulated in NSCLC and played a tumor-suppressive role in this disease by inhibiting cancer cell stemness. We also proved that WT1-AS might achieve its regulatory roles in cancer cell stemness through the down-regulation of TGF-β1.

Previous studies have extensively investigated the roles of WT1-AS in different types of cancers. Du et al. found that WT1-AS was down-regulated in gastric cancer, and the reduced expression levels of WT1-AS were responsible for the increased rate of cancer cell proliferation and invasion [10]. WT1-AS was also down-regulated in cervical cancer, and the over-expression of WT1-AS led to inhibited aggressiveness of cancer cells, indicating that WT1-AS may serve as a target for the treatment of cervical cancer [11]. In hepatocellular carcinoma, WT1-AS down-regulated WT1 to induce the apoptosis of cancer cells [12]. All those previous studies focused on the effects of WT1-AS on different aspects of cancer cell behaviors. Our study focused on cancer stemness, which correlates with cancer cell differentiation and aggressive nature. Stemness is the capacity of cells to perpetuate their lineage and produce differentiated cells. Stemness of a cell determinates its proliferation and regeneration [13, 14]. In effect, inhibition of cancer cell stemness results in inhibited cancer recurrence and metastasis [13, 14]. In the present study, we showed that WT1-AS over-expression resulted in decreased NSCLC cell stemness. Our study revealed a new aspect of the function of WT1-AS in cancer biology.

TGF-β signaling participates in many aspects of cancer development and progression, such as epithelial-to-mesenchymal transition [15], cancer cell proliferation and apoptosis [16], and cancer cell stemness [17]. Our preliminary microarray data revealed an inverse correlation between TGF-β1 and WT1-AS across NSCLC tumor specimens (data not shown). Our study confirmed the enhancing effects of TGF-β1 over-expression on NSCLC cell stemness. It is known that the function of TGF-β signaling in cancer biology can be regulated by specific lncRNAs, such as ANCR and NKILA [18, 19]. In the present study, we showed that WT1-AS was likely an upstream inhibitor of TGF-β1 in the regulation of NSCLC cell stemness. This conclusion is made based on the observation that WT1-AS over-expression led to down-regulated TGF-β1 expression (not the other way) and reduced the effects of TGF-β1 over-expression on cancer cell invasion and migration (co-transfection experiment). Our data suggest that over-expression of WT1-AS may be a target for the treatment of NSCLC. However, more clinical studies are needed to confirm our hypothesis further. It is worth noting that TGF-β1 and WT1-AS were not significantly correlated across normal tissues, indicating that the interaction between TGF-β1 and WT1-AS is likely mediated by certain NSCLC-related factors.

It is worth noting that only the CD133 marker was included in this study to measure cell apoptosis, which is a limitation. Our future studies will consist of more apoptotic markers to further confirm our conclusions.

## Conclusion

In conclusion, WT1-AS was down-regulated in NSCLC, and over-expression of WT1-AS may inhibit the stemness of NSCLC cell stemness by down-regulating TGF-β1.

## Data Availability

The analyzed data sets generated during the study are available from the corresponding author on reasonable request.

## Abbreviation

NSCLC: Non-small-cell lung cancer
